# Community pharmacy professionals’ practice in responding to minor symptoms experienced by pregnant women in Ethiopia: results from sequential mixed methods

**DOI:** 10.1186/s40545-022-00427-x

**Published:** 2022-04-06

**Authors:** Asnakew Achaw Ayele, Md Shahidul Islam, Suzanne Cosh, Leah East

**Affiliations:** 1 School of Health, Faculty of Medicine and Health, University of New England, Armidale, 2351 Australia; 2 Department of Clinical Pharmacy, School of Pharmacy, College of Medicine and Health Science, University of Gondar, Gondar, Ethiopia; 3 School of Psychology, Faculty of Medicine and Health, University of New England, Armidale, 2351 Australia; 4 Hunter New England Health, Armidale, 2350 Australia

**Keywords:** Maternal health, Community pharmacy professionals, Minor symptoms, Pregnancy, Community pharmacy, Community drug retails outlet

## Abstract

**Background:**

In countries with limited access to healthcare services, community pharmacists’ management of minor symptoms experienced by pregnant women could be beneficial in terms of alleviating the burden of other health professionals and cost of services. However, evidence is limited regarding the practice of community pharmacy professionals in responding to minor pregnancy-related symptoms more generally, particularly in Ethiopia.

**Objective:**

The aim of this study was to evaluate actual and self-reported practice of community pharmacists in the management of minor symptoms during pregnancy in Ethiopia.

**Methods:**

A sequential mixed method study using self-reported survey from 238 community pharmacists followed by 66 simulated client visits was conducted from March to July 2020 in six towns of the Amhara regional state in Ethiopia. Independent samples *t*-test and one-way Analysis of Variance was used to test the mean difference of practice score among subgroups of study participants.

**Results:**

The self-reported survey showed that most community pharmacist would ‘always’ gather most symptom-related information particularly about ‘duration of symptoms,’ ‘frequency of symptoms,’ and ‘gestational age’ and provide medication-related information on ‘how to use the medication’ and ‘duration of use.’ The highest mean practice scores were observed in relation to information gathering about ‘gestational age’ and information provision on ‘how to use the medication.’ In contrast, the lowest mean practice scores were observed in relation to information gathering about ‘weight of the woman’ and information provision on ‘dosage form.’

However, the actual practice, as revealed by the simulated client visits, demonstrated that most community pharmacists would rarely gather symptom-related information nor provide medication-related information. In addition, dispensing of non-prescribed medications to pregnant women was also common. The extent of self-reported practice differed among subgroups of study participants.

**Conclusions:**

This study highlights extent of practice of community pharmacy professionals during the management of minor symptoms in pregnancy in Ethiopia. Discrepancies of results between self-reported and actual practices of community pharmacy professionals were observed. The inadequate actual practice of symptom-related information gathering and medication-related information provisions needs considerations of implementing interventions to minimize potential harms.

**Supplementary Information:**

The online version contains supplementary material available at 10.1186/s40545-022-00427-x.

## Background

Due to the physiological changes, symptoms during pregnancy are highly prevalent and can be exacerbated by existing pre-pregnancy conditions [1, 2]. Minor symptoms are common uncomplicated conditions, usually self-limiting, and are often treated without the need of a medical doctor [3]. Pregnant women often suffer from various types of minor symptoms, including but not limited to back pain, nausea and vomiting, headache, vaginal itching, leg pain, indigestion, and diarrhea which can all be managed by a community pharmacist [4, 5]. However, minor symptoms have become a major source of morbidity and mortality due to lack of attention in the maternal health agenda across third world countries [1]. Evidence suggests that the high prevalence of minor symptoms during pregnancy causes significant negative impact on productivity, quality of life, and overall wellbeing [6, 7]. For example, untreated minor symptoms during pregnancy could potentially cause negative adverse effects on pregnant women, the fetus, and subsequently on the newborn. Considering this, literature recognizes the need of treatment for minor symptoms during pregnancy [1]. However, there is a concern related with management of minor symptoms from multiple perspectives, such as limited resources, healthcare burden, and health care delivery costs, especially in countries with low resource settings, such as Ethiopia.

Given the critical shortage of health professionals in Ethiopia, burden associated with management of minor symptoms during pregnancy can be alleviated using community pharmacist-based maternal health service delivery [8–11]. Further, their easily accessibility in the community makes them preferable for clients as a provider of primary health care and medication [12, 13]. Findings from a recent systematic reviews also show that community pharmacy professionals have been involved in providing various types of maternal health service, including management of minor symptoms [9, 10]. In addition, the cost effectiveness of community pharmacists’ management of minor symptoms has been also established in published evidence around the world particularly in developed countries [8, 14, 15].

Although the potential benefit of community pharmacist-based maternal health service provision, such as management of minor symptoms during pregnancy, is unequivocal, the practice needs to be as per the recommended guidelines [16, 17]. During the management of minor symptoms, community pharmacists are supposed to gather comprehensive symptom-related information and provide adequate medication-related information to their clients. However, concerns have been raised regarding the adequacy of information gathered and provided by community pharmacy professionals during management of minor symptoms [18]. For example, poor information gathering practice about duration and frequency of symptoms and inadequate medication-related information provision, such as side effects, have been reported in previous studies conducted in some countries [19, 20] and in Ethiopia [21].

There is minimal published evidence that shows the practice of community pharmacy professionals particularly in responding to minor symptoms during pregnancy globally and especially in Ethiopia. Further, factors linked with poor symptom-related information gathering and inadequate provisions of medication-related information by community pharmacy professionals during the management of minor symptoms have been rarely investigated. Few studies reported interest from customers side, counselling time, communication skills, educational qualifications, and utilization of standard guidelines have been cited as determinates of community pharmacy professionals’ practice during responding to minor symptoms and self-mediations [22–25].

Although evidence is available regarding the practice of community pharmacy professionals in the management of minor symptoms, most of the published articles are based on the general population with limited information regarding management for pregnant women perspective both globally and in Ethiopia. Considering the potential risks of medication use during pregnancy, special attention is needed to pregnant women who are requesting medications for their minor symptoms in community pharmacy. Therefore, evidence is needed regarding community pharmacy professionals practice when responding to minor symptoms in pregnant women, in order to support good practice and promote wellbeing in pregnancy. In pharmacy practice research, it is recommended to conduct mixed methods of self-reported survey and simulated client visit approach [26]. This is because the addition of simulated client visit (post-survey) has been considered methodologically rigorous and robust as it will allow participants to be observed in their natural environment thereby to investigate the actual and consistency of practice [26].

Therefore, the aim of this study was to investigate both the self-reported and the actual practice of community pharmacy professionals in responding to minor symptoms experienced by pregnant women using a sequential mixed method approach (self-reported survey followed by simulated client visits). In addition, this study was also identified factors liked with extent of community pharmacy professionals practice during management of minor symptoms during pregnancy. The evidence generated from this study has the potential to contribute to knowledge and evidence gaps by informing the current practice of community pharmacy professionals in the management of minor symptoms during pregnancy more generally, as well as Ethiopia in particular.

## Methods

### Study design

A two-phase sequential mixed method study was employed to address the aim of this study. For the purposes of this study, a quantitative self-reported survey (phase one) and simulated client visit (phase two) were employed. The first phase of this study was a self-reported survey. The purpose of the self-reported survey was to investigate the current self-reported practice of community pharmacy professionals in responding to minor symptoms for pregnant women in Ethiopia. Self-reported practice obtained in phase one may be subject to reporting bias and may not fully reveal the actual practice. Thus, it is recommended that simulated client visits are undertaken post self-reported survey to verify self-reported practice [26, 27]. Therefore, we used a simulated client visit method in the second phase after the self-reported survey had been completed to investigate the actual practice of community pharmacists in the management of minor symptoms during pregnancy and to compare the findings with the self-reported survey. The simulated visits were conducted in the Community Drug Retail outlets (CDROs) that participated in the survey and results were matched to evaluate the consistency of practice. In healthcare research, a simulated patient also known as a simulated client is an individual trained to act as a real patient in order to simulate a set of symptoms or health problems [26]. Simulated patients have been successfully utilized for the evaluation of health care professionals’ practice because it allows for professionals to be observed in their natural environment, uninfluenced by the awareness that their behavior is being observed [27].

### Study area and samples

This study was conducted with community pharmacy professionals practicing in CDROs of six randomly selected cities of Amhara regional state, Ethiopia. From a total of 11 zonal cities found in Amhara regional state, six cities were randomly selected (Selected cities: Debre Markos, Gondar, Dessie, Bahir Dar, Debre Tabor, and Debre Birhan). The sample size needed to conduct the survey was determined using the simple proportion formula assuming the 5% margin error, 95% confidence level, and 50% response distribution. Considering 852 active CDROs found in Amhara regional state as of November 2019, we used correction formula, and thus, the final sample size was 264. Therefore, the survey was conducted among 264 community pharmacists (one pharmacist per CDRO) who had been working in the selected cities and CDROs. The survey included participants who work in any of the selected cities in the region as a qualified and licensed pharmacy professional for at least 3 months. We excluded pharmacy students who are practicing as interns in the selected cities and CDROs. Sampled participants were recruited randomly from the list of selected CDROs in each study site.

This study was undertaken as part of a larger study aimed at evaluating community pharmacy professionals practice in maternal (part one) and child (part two) health services. There is no universal standard on the sample size calculation for simulated client visits. However, we aimed at having data from half of the participants involved in the survey. Thus, a minimum of 132 CDROs were needed for post-survey simulated client visits for both parts of the study. Again the 132 CDROs were divided into two (66 CDROs for each part). In part one (maternal health), our simulated client visit consisted of two case scenarios (back pain and nausea and vomiting). That means a total of 66 CDROs were further divided into two cases (33 CDROs for each case). The results of the child health simulated visit have not been included in this study and will be reported separately as the objective was different.

### Data collection tools

#### Phase one––self-reported survey

This survey was conducted using a self-administered structured survey (Additional file 1). The survey was designed to assess self-reported extend of practice of community pharmacy professionals in relation to maternal health care. The survey items were developed from previously published literature in the field relevant to our objectives [5, 28–30]. The content of the survey was reviewed by experts in pharmacy practice research (two clinical pharmacists and one public health researcher). The feedbacks from the experts were discussed by the research team for appropriateness and inclusion in the final survey tool. After feedbacks from the expert incorporated, the survey questionnaires were further pilot tested with 10 community pharmacy professionals at two different study sites––Gondar and Bahir Dar (data were not included in the final analysis). Feedback from the pilot testing was used to improve wording and clarity of items. Finally, the survey questionnaires were revised by the research team after incorporation of the suggested minor content and wording modifications by pilot participants. The survey questionnaires consist of three parts. The first part included data on participants’ demographic characteristics, such as gender- and professional-related information, such as educational achievements, professional licensing level, work experience in CDROs, responsibility in CDROs, and training experience about maternal health services. The second part of the survey included items designed to assess extent of practice of community pharmacy professionals in management of minor symptoms in pregnancy. This part of the survey tool has two sections. The first section contains 8 items relating to symptom-related information gathering practice. Items were scored on a 4-point Likert scale indicating frequency of the practice (‘always,’ ‘usually,’ ‘sometimes,’ and ‘never’), with higher scores indicating greater frequency. Section two included 10 items related to medication-related information provision practice. Items were scored on the same 4-point Likert scale indicating frequency of the practice. Internal consistency for the items related to practice (part two) was *α* = 0.83 ‘for symptoms related information gathering’ and α = 0.81 for ‘medication related information provision,’ demonstrating good internal consistency. The last part (part three) of the survey tool focused on practice of community pharmacists for specific cases scenarios (back pain, and nausea and vomiting). This section of the survey was designed to match the results obtained from the self-reported practice with the simulated case scenarios for the selected cases. In this part, an item intended to assess most common types of minor symptoms presenting to CDROs by pregnant women was also included. Participants were asked to tick from the list of minor symptoms they have been requested frequently by pregnant women. Space was also provided to enable participants to write minor symptoms not included in lists.

#### Phase two––simulated client visit methods

Two case scenarios (back pain, and nausea and vomiting) were designed to assess the response of pharmacy professionals working in CDROs for request of treatments. These symptoms have been used for simulated cases in previous research [31]. The case scenarios were designed to assess symptom-related information gathering and medication-related information provision practice. Information related with the practice (symptom-related information and medication-related information provision practice) was captured using prepared data extraction format designed for simulated client visit outcomes (Additional file 2). Details about the case scenarios and the scripts played during the visit are shown in Table 1

**Table 1:** Case scenarios to evaluate the actual practice of community pharmacy professionals' in providing health services for pregnant women focusing on responding to minor symptoms

*Scenario 1: back pain*	
A male Simulated Client (SC) asked the pharmacy staff to give him a medication for his wife with 8th month of pregnancy complaining of back pain. The community pharmacy professional was expected to ask information about the symptoms and rule out other medical conditions and advise the SC to take safe anti-pain, such as paracetamol, if insufficient advice to visit the nearby hospital if the symptoms persist. The community pharmacy professional was also expected to give adequate medication-related information as per to the standard dispensing practice	
The community pharmacy professional was given the following information when asked	✓ No previous or current medical conditions other than the complaint of back pain
	✓ She was at 8th month of pregnancy
	✓ The pain started 1 week ago
	✓ The pain was happening every day and gets worse at night
	✓ She has not taken any medications before
	✓ She was 27 years old and weighed 56 kg
	✓ She did not visit any health facility for the case
*Scenario 2: moderate nausea and vomiting*	
The SC was pregnant woman in the 9th weeks of pregnancy complains of moderate nausea and vomiting. The community pharmacy professional was expected to ask detailed symptom-related information and rule out other medical conditions and advise the SC to take safe antihistamines and or non-pharmacological interventions, such as herbal remedies (example ginger), if the symptom persists advise to visit the nearby hospital. The community pharmacy professional was also expected to deliver adequate medication-related information	
The community pharmacy professional was given the following information when asked	– No previous or current medical conditions other than the complaint of nausea and vomiting
	– She was in first trimester of pregnancy
	– The pain started 1 week ago and was happening every day
	– She has not taken any medications before
	– She was 27 years old and weighed 56 kg
	– She did not visit any health facility for the case
All information given by the community pharmacy professional was recorded using structured data recording format as soon as the SC left the pharmacy (Additional files 1, 2)	

### Ethics approval

Ethics approval was granted from University of New England Human Research Ethics Committee (HE20-021). Written consent was also obtained from each respondent for the simulated client visit. The consent was obtained during the first phase of the study (self-reported survey). The participants were informed about the purpose of the study and a visit would occur but not told the schedule of the visit.

### Data collection procedures

#### Self-reported survey (phase one)

Trained data collectors (pharmacists) were assigned to collect both self-report surveys and undertake the simulated client visits. Random lists of CDROs in each study city were prepared. The data collectors first approached the selected CDROs and asked anyone in the pharmacy to identify the pharmacy professionals who are licensed and authorized to work in the CDRO. Once they identified the licensed pharmacist, the data collectors invited them to participate by providing the participants’ information sheet followed by the consent form. Following consent, the survey was given to participants for completion after the data collector had left. The completed surveys were collected from CDROs at a later time.

#### Simulated client visits (phase two)

The simulated client visit was conducted from those who completed the self-reported survey and who provided consent to be visited. A total of 66 CDROs have been randomly selected from those who competed the self-reported survey. The simulated clients were pharmacy professionals who had not participated in the survey data collection to avoid social desirability bias. Each of the simulated clients were given a specific scenario to play. Detailed instruction and customized training were delivered about how to act the scenario (the simulated case) by the first author. The simulated client visit was conducted 3 months after consent was provided. The self-reported response and the simulated client visit data were matched to observe the difference in practice of the selected case scenarios.

### Data analysis

The quantitative data were cleaned, entered, and analyzed using Statistical Package for the Social Sciences (SPSS), version 26 (SPSS, Inc., Chicago, IL, USA). Categorical variables were presented as frequencies and percentages. Mean and standard deviation were used to summarize continuous variables. Independent sample *t*-test and one-way analysis of variance (ANOVA) with Bonferroni test (post hoc) analyses were used to compare the mean difference of practice scores and to identify factors liked with community pharmacy professionals’ practice in management of minor symptoms during pregnancy among subgroups of participants’ characteristics, such as gender, work experience, educational qualifications, licensure level, CDROs setting types, and training experience about maternal health service. Level of statistical significance was declared at a two-sided *p*-value ≤ 0.05. The data from self-reported response and simulated client visits for each cases scenarios were compared based on number and percentage of participants who have gathered symptom-related information and provided medication-related information.

## Results

### Characteristics of the participants

Of the 264 community pharmacy professionals approached in the selected study sites, 240 participants returned the questionnaires. Following screening for completeness, two questionnaires were excluded due to being partially incomplete. Finally, a total of 238 completed questionnaires were included for the data analysis with a response rate of 90%. Details of characteristics of the community pharmacy professionals are included in Table 2.

**Table 2:** Characteristics of the community pharmacy professionals

Characteristics of the community pharmacy professionals	Total (*n* = 238)
Characteristics	*n* (%)
Sex	
Male	117(49.2)
Female	121(50.8)
Educational qualification in pharmacy	
Diploma in pharmacy	130(54.6)
Bachelor of pharmacy (BPharm)	91(38.2)
Master of pharmacy (MSc)	17(7.1)
Work experience in years (mean, *SD*)	6.05 ± 5.49
Work experience in category (based on years required for the next licensure level)	
Less than 5 years	130(54.6)
5–10 years	40(16.8)
Greater than 10 years	68(28.6)
Licensure by regulatory authority	
Druggist/Pharmacy technician	96(40.3)
Junior pharmacist	48(20.2)
Senior pharmacist/druggist	45(18.9)
Chief pharmacist	19(8.0)
Expert pharmacist	30(12.6)
Facility (community drug retails outlet) type	
Drug store	106(44.5)
Pharmacy	132(55.5)
Responsibility in the community drug retails outlet	
Owner	96(40.3)
Employed	142(59.7)
Have you received any in-services training regarding maternal and or child health services delivery in community drug retails outlets?	
Yes	42(17.6)
No	196(82.4)

### Practice of community pharmacy professionals in response to minor symptoms for pregnant women

Mean level of practice and percentage distribution for each item of both symptom-related information gathering and medication-related information provision is shown in Table 3.

**Table 3:** Percentage distribution and mean practice score of community pharmacy professionals in management of minor symptoms for pregnant women

Items	Response = *n* (%)				Mean practice level	
	Always	Often	Sometimes	Never	Mean	*SD*
A. Considering your role in management of minor symptoms during pregnancy: how often do you gather the following symptom-related information before providing a treatment?						
Duration of symptoms	112 (47.1)	83(34.9)	42(17.6)	1(0.4)	3.29	.765
Frequency of symptoms	97(40.8)	83(34.9)	54(22.7)	4(1.7)	3.15	.826
Comorbidity	81(34.0)	74(31.1)	76(31.9)	7(2.9)	2.96	.883
Age of the woman	76(31.9)	58(24.4)	93(39.1)	11(4.6)	2.84	.934
Gestational age/Trimester/	129(54.2)	58(24.4)	46(19.3)	5(2.1)	3.31	.853
Weight of the woman	45(18.9)	57(23.9)	108(45.4)	28(11.8)	2.50	.931
Previous medical conditions	69(29.0)	81(34.0)	81(34.0)	7(2.9)	2.89	.860
Previous medication history and current medication, allergy history	85(35.5)	78(32.8)	67(28.2)	8(3.4)	3.01	.881
Overall mean of practice score of information gathering					*23.9370*	*4.67693*
B. Considering your role in responding to symptoms for pregnant women: how often do you inform the clients about the following medication-related information when dispensing a medication?						
Name of the medication	91(38.2)	60(25.2)	80(33.6)	7(2.9)	2.99	.916
Purpose/use of medication	141(59.2)	85(35.7)	10(4.2)	2(0.8)	3.53	.620
Dosage form	89(37.4)	61(25.6)	77(32.4)	11(4.6)	2.96	.940
Dose	154(64.7)	37(15.5)	44(18.5)	3(1.3)	3.44	.833
Information on how to use the medication and its application	205(86.1)	31(13.0)	1(0.4)	1(0.4)	3.85	.403
Duration of use	202(84.9)	32(13.4)	4(1.7)	0(0.0)	3.83	.417
Side effect	92(38.7)	77(32.4)	64(26.9)	5(2.1)	3.08	.858
Drug interaction	106(44.5)	73(30.7)	58(24.4)	1(0.4)	3.19	.819
Importance of compliance/adherence	170(71.4)	44(18.5)	23(9.7)	1(0.4)	3.61	.677
Storage conditions	135(56.7)	66(27.7)	34(14.3)	3(1.3)	3.40	.777
Overall mean practice of medication-related information provision					*33.8739*	*4.58037*

#### Symptom-related information gathering practice

As observed from Table 3 section A, the most frequent practice regarding symptom-related information gathering was regarding ‘gestational age/trimester’ (mean = 3.31, *SD* = 0.853) with more than half (54.2%) of community pharmacy professionals reporting they have ‘always’ been gathering symptom-related information about 'gestational ag'e or 'trimester'. The second highest mean practice score was observed from community pharmacy professionals practice regarding symptom-related information gathering about ‘duration of symptoms’ (mean = 3.29, *SD* = 0.765). Approximately 47.1% of the study participants reported that they have been ‘always’ gathering symptom-related information about ‘duration of symptoms.’ In contrast, the lowest mean level of practice was observed regarding ‘weight of the woman’ (mean = 2.50, *SD* = 0.931). Most study participants (45.4%) reported that they asked 'sometimes’ information about ‘weight of the woman.’ In addition, about 12% community pharmacy professionals reported that they never asked the 'weight of pregnant women' before providing treatment for minor symptoms. The participants also scored the second lowest mean practice regarding information gathering about ‘age of the woman’ (mean = 2.84, *SD* = 0.934). While 39.1% of community pharmacy professionals reported that they ‘sometimes’ asked about 'age'-related information, only 31.9% them asked this information ‘always.’ Details of symptom-related information gathering practice is available in Table 3 section A.

#### Medication-related information provision practice

A majority of the participants reported that they have ‘always’ been providing most of the medication-related information. The highest mean practice scores were observed in relation to ‘information on how to use the medication and its application’ (mean = 3.85, *SD* = 0.403) and ‘duration of use’ (mean = 3.83, *SD* = 0.417). The percentage distribution in Table 3 section B also showed that a large proportion of the participants were “always” providing medication-related information regarding ‘information on how to use the medication and its application’ (86.1%) and ‘duration of use’ (84.9%). In contrast, the lowest mean practice scores were observed in ‘dosage form’ (mean = 2.96, *SD* = 0.940) and ‘name of medication’ (mean = 2.99, *SD* = 0.916). Only, 37.4% and 38.2% of the participants were reported they have ‘always’ been providing ‘dosage form’ and ‘name of medication’-related information, respectively. The details of community pharmacy professionals practice in providing medication-related information are found in Table 3 section B.

### Total practice scores among different characteristics of respondents

#### Mean difference of symptom-related information gathering practice score

As presented in Table 4, a significant difference of symptom-related information gathering practice was observed between level of responsibility in the pharmacy, with ‘owner’ and ‘employee’ with owners engaging in more frequent practice (*p*-value = 0.003). The ANOVA test result also showed that significant mean differences were observed across participants educational qualification in pharmacy, work experience in years, and licensure level by regulatory authority. As observed in Table 5, the post hoc analysis revealed that, in comparison with participants who had a ‘master’s degree in pharmacy’ (mean = 26.35), those who had educational qualification of ‘Diploma in Pharmacy’ (mean = 23.39) reported fewer mean symptom-related information gathering practice (*p*-value = 0.042).* A* significant difference was also observed between participants with greater than 10 years of experience and less than 5 years of experience in CDRO (*p*-value ≤ 0.001). Similarly, a statistically significant difference was observed based on participants’ licensure level. Community pharmacy professionals *with* licensure level ‘chief pharmacist’ reported more frequent symptom-related information gathering practice than professionals with licensure level of ‘druggist/pharmacy technician’ (*p*-value = 0.001)* and junior* pharmacist (*p*-value = 0.011). Significant differences were not observed in relation to medication-related information provision among the subgroups of community pharmacy professionals in this study.

**Table 4:** Practice of community pharmacy professionals among different subgroups of study participants: independent sample *t*-test and one-way ANOVA

Demographic and related characteristics	Categories	Symptom-related information gathering practice		*p*-value Levene’s test for equality of variances	Medication-related information provision practice		*p*-value Levene’s test for equality of variances
		Mean, *SD*	*p*-value		Mean, *SD*	*p*-value	
Sex	Male	24.43 ± 4.77	0.106	0.310	34.21 ± 4.34	0.261	0.243
	Female	23.45 ± 4.54			33.54 ± 4.79		
Educational qualification in pharmacy	Diploma in pharmacy	23.39 ± 4.76	0.034*	0.362	33.76 ± 4.750	0.835	0.505
	Bachelor of pharmacy (BPharm)	24.26 ± 4.49		S	33.91 ± 4.47		
	Master of pharmacy (MSc)	26.35 ± 4.22			34.47 ± 3.93		
Work experience in years	Less than 5 years	22.96 ± 4.51	0.001*		33.43 ± 4.99	0.080	0.011
	5–10 years	24.17 ± 4.50		0.777	33.52 ± 4.24		
	Greater than 10 years	25.64 ± 4.64			34.92 ± 3.76		
Licensure by regulatory authority	Druggist/Pharmacy technician	22.92 ± 4.62	0.001*	0.491	33.69 ± 4.78	0.792	0.732
	Junior pharmacist	23.41 ± 4.48			33.35 ± 4.78		
	Senior Pharmacist/Druggist	25.08 ± 4.73			34.20 ± 4.52		
	Chief pharmacist	27.47 ± 3.74			34.47 ± 4.32		
	Expert pharmacist	24.03 ± 4.39			34.40 ± 3.93		
Facility type	Drug store	23.33 ± 4.37	0.073	0.146	33.27 ± 4.31	0.070	0.221
	Pharmacy	24.42 ± 4.87			34.35 ± 4.74		
Responsibility in the pharmacy	Owner	25.03 ± 4.54	0.003*	0.759	34.23 ± 4.18	0.312	0.115
	Employed	23.19 ± 4.63			33.62 ± 4.82		
In-services training received	Yes	24.42 ± 4.35	0.454	0.689	34.23 ± 4.94	0.571	0.170
	No	23.83 ± 4.74			33.79 ± 4.50		

*The mean difference is significant at the 0.05 level

**Table 5:** Post hoc analyses of factors influencing community pharmacy professional’s practice

Symptom-related information gathering practice				
Variables	Factors	Group	Mean difference	*p*-value
Educational qualification in pharmacy	Diploma in pharmacy	Master of pharmacy (MSc)	− 2.96063*	0.042*
Work experience in years	Greater than 10 years	Less than 5 years	2.67783*	< 0.001*
Licensure by regulatory authority	Chief pharmacist	Druggist/Pharmacy technician	4.54660*	0.001*
		Junior pharmacist	4.05702*	0.011*

*The mean difference is significant at the 0.05 level

### Common minor symptoms presented in community pharmacies by pregnant women

**Fig. 1:**
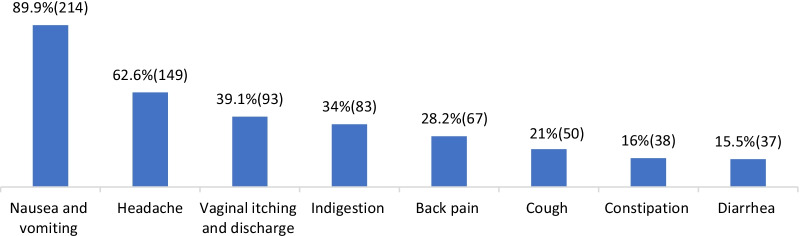
Commonly presented minor symptoms by pregnant women at community drug retails outlets. Key: Percentage: Percent of community pharmacy professionals from the total (238) who reported each symptom. The numbers in the brackets are number of community pharmacy professionals who reported each symptom

Nausea and vomiting (89.9%), headache (62.6%), and vaginal itching (39.1%) were the top three common minor symptoms why pregnant women sought treatment from community pharmacists. The participants also reported that they had been requested to provide treatment for indigestion (34.9%), back pain (28.2%), and cough (21%). Constipation (16%) and diarrhea (15.5%) were reported as a common minor symptom by small proportions of the study participants. Common minor symptoms are presented in Fig. 1.

### Simulated clients visit and self-reported matching results

#### Symptom-related information gathering practice

For both case scenarios (back pain, and nausea and vomiting), the self-reported survey response showed that most of the participants reported that they asked symptom-related information about ‘duration of symptom,’ ‘frequency of symptoms,’ ‘comorbidity,’ ‘gestational age,’ and ‘previous medical conditions.’ However, in actual practice, the simulated client visit showed that the proportion of the participants who asked symptom-related information was different and low compared to the self-reported response. For example, in case scenario ‘back pain,’ the percentages of community pharmacy professionals who reporting asking symptom-related information in the self-report compared with practice observed in the simulated client visit were as follows: ‘duration of symptoms’ (78.8% vs 45.5%), ‘frequency of symptoms’ (63.6% vs 54.5%), ‘comorbidity’ (60.6% vs 27.3%), ‘gestational age’ (87.9% vs 57.6%), and ‘previous medical conditions’ (48.5% vs 33.3%), respectively. This indicates that the actual practice in relation with symptom-related information gathering practice is different from the self-reported survey response. Result comparisons of the self-reported and simulated client visit regarding symptom-related information gathering practice are presented in Table 6.

**Table 6:** Self-reported survey and simulated client visit data matching results

A. Case scenario one: back pain *n* = 33					
Symptom-related information items	Results obtained (information gathered)		Medication-related information items	Results obtained (information provided)	
	Self-reported survey *n* (%)	Simulated clients visit *n* (%)		Self-reported survey: *n* (%)	Simulated clients visit *n* (%)
Duration of symptoms	26(78.8)	15(45.5)	Name of the medication	11(33.3)	11(34.4)
Frequency of symptoms	21(63.6)	18(54.5)	Purpose of medication	23(69.7)	15(46.9)
Comorbidity	20(60.6)	9(27.3)	Dosage form	10(30.3)	10(31.3)
Age of the woman	15(45.5)	4(12.1)	Dose	20(60.6)	15(46.9)
Gestational age	29(87.9)	19(57.6)	How to use the medication	30(90.9)	22(68.8)
Weight of the woman	10(30.3)	3(9.1)	Duration of use	27(81.8)	18(56.3)
Previous medical conditions	23(69.7)	11(33.3)	Side effect	23(69.7)	3(9.4)
Previous medication history and current medication, allergy Hx	16(48.5)	6(18.2)	Drug interaction	21(63.6)	1(3.1)
			Importance of adherence	25(75.8)	4(12.5)
			Storage conditions	27(81.8)	1(3.1)

B. Case scenario two: nausea and vomiting *n* = 33					
Symptom-related information gathering	Self-reported survey	Simulated client visit	Medication-related information provision	Self-reported survey	Simulated client visit
Duration of symptoms	29(87.9)	23(69.7)	Name of the medication	12(36.4)	10(31.3)
Frequency of symptoms	22(66.7)	19(57.6)	Purpose of medication	25(75.8)	18(56.3)
Comorbidity	21(63.6)	3(9.1)	Dosage form	11(33.3)	7(21.9)
Age of the woman	11(33.3)	0(0.0)	Dose	26(78.8)	13(40.6)
Gestational age	27(81.8)	22(66.7)	How to use the medication	31(93.9)	16(50.0)
Weight of the woman	7(21.2)	1(3.0)	Duration of use	24(72.4)	17(53.1)
Previous medical conditions	22(66.7)	11(33.3)	Side effect	18(54.5)	1(3.1)
Previous medication history and current medication, allergy	22(66.7)	3(9.1)	Drug interaction	20(60.6)	2(6.3)
			Importance of adherence	24(72.7)	2(6.3)
			Storage conditions	22(66.7)	1(3.1)

#### Medication-related information provision practice

Regarding medication-related information provision, in both case scenarios, most of the participants from the self-reported survey group reported that they provided most of the information listed except ‘name of medication’ and ‘dosage form.’ However, what was seen in the actual practice was quite different. In the simulated client visit, most of the participants did not provide medication-related information in most of the assessed areas. To be more specific, information about ‘side effect,’ ‘drug interaction,’ ‘importance of adherence,’ and ‘storage conditions’ were among medication-related information provided by very few participants in the simulated client visit in both case scenarios*.* Similarly, with the symptom-related information gathering practice, the actual and the self-reported practice of medication-related information provision was found to be different. Result comparisons of the self-reported and simulated client visit regarding medication-related information provision practice are presented in Table 6.

### Community pharmacy professionals’ decision and the characteristics of the medications given during the simulated client visit

In both case scenarios, only one community pharmacy professional did not give medication to the simulated client. Paracetamol (40.6%) and tramadol (37.5%) were the most dispensed medications to ‘back pain’ simulated client. Metoclopramide (40.6%) and ondansetron (21.9%) were dispensed by the majority of the community pharmacy professionals to the ‘nausea and vomiting’ simulated clients. From the dispensed medications to the ‘back pain’ simulated clients, one medication (tramadol) was not listed as OTC (Over the Counter) by Ethiopian Food and Drug Authority (EFDA) and is not allowed to be sold without prescriptions in CDROs. Similarly, among the medications dispensed to ‘nausea and vomiting’ simulated clients, three medications (ondansetron, metoclopramide, and meclizine) were not listed as OTC medications by the EFDA. This indicates that dispensing of medications without prescription to pregnancy women during management of minor symptoms was also reasonably common (Table 7).

**Table 7:** Characteristics of medications given during the simulated client visits

Name of medications dispensed	(*n*, %)	OTC* category
A. Case scenario one: back pain, *n* = 32		
Paracetamol	13(40.6)	Yes*
Tramadol	12(37.5)	No*
Diclofenac	7(21.9)	Yes*
Ibuprofen	1(3.1)	Yes*
B. Case scenario two: nausea and vomiting, *n* = 32		
Ondansetron	7(21.9)	No*
Chlorpheniramine	4(12.5)	Yes*
Metoclopramide	13(40.6)	No*
Meclizine	6(18.8)	No*
Diclofenac	1(3.1)	Yes*
Multivitamin complex	1(3.1)	Yes*

OTC*: Over the Counter—the category was determined as per Ethiopian Food and Drug (EFDA) administration OTC list. Yes* indicates that the medication is listed as OTC and No* indicates that the medication is not listed as OTC and not allowed to sell without prescription

## Discussion

This study evaluated the actual and self-reported practice of community pharmacy professionals in responding to minor symptoms for pregnant women in Ethiopia. In the self-reported survey for general practice, most of the community pharmacy professionals reported that they either ‘always’ or ‘often’ gathered ‘symptoms related information’ particularly about duration of symptoms, frequency of symptoms, and gestational age/trimester. In contrast, community pharmacy professionals were less frequently gathering information related to age of the woman, weight of the woman, and previous medical conditions. Further, in the present study, most of the community pharmacy professionals reported that either ‘always’ or ‘often’ provided medication-related information about purpose of medication, how to use the medication, duration of use, importance of adherence, dose, storage conditions, side effect, and drug interaction. However, information about dosage form and name of the medication were reported to have been provided less frequently. Overall, the self-reported survey results of this study suggest that the frequency of symptom-related information gathering and medication-related information provision practice in response to minor symptoms to pregnant women seem adequate for most of the components, but insufficient for some of the information types, such as age of woman, weight of woman, previous medical conditions, name of medication, and dosage form. Previous research has shown that the types of information gathered and medication-related information provided are inconsistent in different settings and countries [19, 24, 32, 33]. However, information about age, weight, and previous medication history were rarely gathered and information about dosage form and name of medication were less frequently provided by community pharmacy professionals during management of minor alignments [19, 32]. Inadequate gathering and provision of this information generally could lead to misdiagnosis of symptoms and recommendation of inappropriate medications [34].

Although community pharmacy professionals have been reported to gather symptom-related information, their extent of practice differed among different characteristics of the participants, such as responsibility in the CDROs, educational qualifications, work experience, and licensure level. For example, frequency of symptom-related information gathering among community pharmacy professionals who were employees in the CDRO was less than owners of the CDROs. Although this difference has not been seen in literatures, this might be possibly employees may not fell the freedom of using unlimited time for symptom-related information gathering practice to cover all components of the information.

In this study, it is also noted that community pharmacy professionals with lower educational qualification in pharmacy (diploma in pharmacy) were linked to infrequent symptom-related information gathering. It is not surprising that community pharmacy professionals with a lower education level did not frequently gathered symptom-related information as the knowledge and skills gained might be more limited [25]. However, empowering community pharmacy professionals with additional tailored training focusing on the importance of gathering comprehensive symptom-related information during the management of minor ailments in pregnancy could improve the practice [35, 36]. Similarly, having less than 5 years of work experience in CDRO was linked with infrequent symptom-related information gathering practice. This indicates that extensive work experience is needed to improve the frequency of symptom-related information gathering practice during management of minor symptoms in pregnant women, as it is a requirement to improve the quality of services and to avoid potential adverse effects of medications that could be happen in pregnancy. Lack of longer work experience has also has been linked with inadequate symptom-related information practice during management minor ailments and self-medications in previous studies conducted elsewhere [19, 37].

Furthermore, community pharmacy professionals who had ‘chief’ level license more frequently gathered symptom-related information compared to those who were ‘pharmacy technician’ and ‘junior pharmacist’ in their licensure level. This could possibly be due to two reasons: (1) the lower education qualification pharmacy technicians had and/or (2) the limited involvement of ‘pharmacy technician’ and ‘junior pharmacist’ in the provision of maternal health service in Ethiopia. To explain the second reason more, in Ethiopia, community pharmacy professionals with ‘pharmacy technician’ and ‘junior pharmacist’ licensure level do have not the mandate to work as main pharmacist, instead they are authorized to work as an assistant pharmacist. This would likely limit the scope of their practice in providing various services, including responding to minor symptoms.

Another important point in this study is inconsistency of results from self-reported survey and simulated client visit in relation to symptom-related information gathering and medication-related information provision practice. The self-report findings for both cases scenarios showed that most of the community pharmacy professionals reported that they have been gathered most of symptom-related information and provided most of medication-related information. However, the simulated client visits data reveal that only a smaller proportion of community pharmacy professionals gathered both symptom-related information and provided medication-related information. Although the findings from the self-reported survey are more favorable than the simulated client visit (actual practice), still there were some types of symptom- and medication-related information less frequently gathered and provided particularly weight of women, age of women, previous medication history, name of medication, and dosage form. Inconsistencies of results between self-reported survey and simulated client visit of community pharmacy professionals in responding to minor symptoms have been reported in previous studies [9, 32, 38]. In general, the actual practice of community pharmacy professionals in responding to minor symptoms for pregnant women was found to be different and poorer practice compared with what was observed in the self-reported survey. In this regard, community pharmacy professionals need to note their professional role and be work with responsibility as per available recommendations [17].

Further, our study identified the non-prescribed dispensing of medications for minor symptoms during simulated client visits. This indicates that, apart from the actual poor practice, community pharmacy professionals have been managing minor symptoms in pregnancy with medications that requires authorized prescriptions. The high rate of dispensing of medications without prescription and self-medication in developing countries, including Ethiopia, is concerning as it has been reported in other studies [21, 39, 40]. In such cases, the benefits of treating minor symptoms in pregnancy in CDROs might not outweigh the risks of taking medications that requires prescription from legally authorized health professionals. Overall, safe use of medications for minor symptoms during pregnancy demands careful consideration of risk–benefit analysis of each treatment [28, 41].

Although community pharmacy professionals have a great role in the management of minor symptoms during pregnancy, the observed actual poor practice of symptom-related information gathering and medication-related provisions is concerning from three perspectives. Firstly, without assessing adequate symptom-related information, community pharmacy professionals might misdiagnose the symptoms and provide inappropriate medications. Secondly, the inadequate information gathering practice by community pharmacy professionals might lead pregnant women to take medications which are risky during pregnancy. Thirdly, the inadequate provision of medication-related information by community pharmacy professionals could lead to adverse effects, medication misuse, non-adherence, and treatment infectiveness [28, 42].

Indeed, poor practice of symptom-related information gathering and medication-related information provision during self-medication and responding to minor symptoms is a worldwide problem as evidenced by a recently published systematic review [32]. However, the factors of inadequate symptom-related information and medication-related provision practice are often multifaceted and need further exploration from the context of the country where community pharmacy professionals are practicing. Therefore, to maximize the benefits of community pharmacy-based management of minor symptoms for pregnant women and to lower the risks of poor symptom-related information gathering and medication-related information provisions, some interventions are needed. For example, empowering of community pharmacy professionals through customized training, development, and implementation of specific guidelines and strict government regulations could help in improving the practice.

## Strength and limitation of the study

To the best of our knowledge, this study was the first in evaluating the practice of community pharmacy professionals in responding to minor symptoms in the context of pregnant women in Africa and Ethiopia using a sequential mixed methods approach in a multi-center study. Social desirability and recall bias in the self-report (phase one) could be mentioned as a limitation in this study. However, our study employed a sequential mixed methods of self-reported followed by a simulated client visit which can verify the actual practice. Further, the addition of simulated client visit method in our study could strength the methodological rigor in evaluating professional practice.

## Implications for policy, future practice, and research

The findings of our study have provided imperative insights regarding the current practice of community pharmacy professionals in the management of minor symptoms in pregnancy in Ethiopia, which can be used as a benchmark for designing and implementing interventions and polices. For example, to improve practice, this study might help in the development of guidelines specific to the management of minor ailments in CDROs in the context of Ethiopia and more broadly. Further, our findings regarding dispensing of non-prescribed medications to pregnant women during the management of minor symptoms needs considerations to avoid the potential harms associated with it. Considering the complexity of reasons for poor pharmacy practice, we also suggest further detailed exploration of factors hindering community pharmacy professionals in management of minor symptoms within the context of pregnant women in Ethiopia.

## Conclusions

Community pharmacy professionals in Ethiopia have been involved in the management of various types of minor symptoms for pregnant women. Although, the self-reported survey identified that community pharmacy professionals have been gathering and providing most components of symptom-related and medication-related information, the actual practice reveals that both symptom-related and medication-related information have been less commonly gathered and provided. Dispensing of non-prescribed medications for the management of minor symptoms during pregnancy also needs regulation from health authorities considering the potential harms of medications use during pregnancy. Responsibility in the CDROs, educational qualifications, work experience, and licensure level were determinates of community pharmacy professionals’ practice related with information gathering in managing of minor symptoms during pregnancy.

## Supplementary Information

**Additional file 1.** Data collection tools -self-reported survey (phase one).

**Additional file 2.** Simulated client visit outcome recording forms.

## Data Availability

The datasets used and/or analyzed during the current study are available from the corresponding author on reasonable request.

## Abbreviations

ANOVA: One-way Analysis of Variance
CDROs: Community Drug Retail outlets
EFDA: Ethiopian Food and Drug Authority
OTC: Over the Counter
SC: Simulated Client
