# Supplementary material for: Community pharmacy professionals’ practice in responding to minor symptoms experienced by pregnant women in Ethiopia: results from sequential mixed methods
Source: J Pharm Policy Pract. 2022 Apr 6;15:29. doi: 10.1186/s40545-022-00427-x (PMC8988357; doi:10.1186/s40545-022-00427-x)
Supplement: Supplementary file 1 — Additional file 1. Data collection tools -self-reported survey (phase one). [file 40545_2022_427_MOESM1_ESM.docx]

**Additional file 1: Data collection tools -self-reported survey (phase one)**

**Part one: Demographic data**

| Items | | Response |
| --- | --- | --- |
| 1 | Gender | [1] Male [2] Female |
| 2 | Educational qualification in Pharmacy | 1. Diploma in pharmacy 2. Bachelor of Pharmacy (BPharm) 3. Doctor of Pharmacy (PharmD) 4. Master of Pharmacy (MSc) 5. Doctor of Philosophy (PhD.) |
| 3 | Year of experience in community pharmacy | ……………months |
| 4 | Licensure by regulatory Authority | 1. Druggist/Pharmacy technician 2. Junior Pharmacist 3. Senior Pharmacist 4. Chief Pharmacist 5. Expert Pharmacist |
| 5 | Location of Community Drug Retail Outlets (CDROs) | 1. Dessie 2. Debre Tabor 3. Bahir Dar 4. Debre Birhan 5. Debre Markos 6. 6Gondar |
| 6 | Responsibility in the CDRO | 1. Owner 2. Employed |
| 7 | Have you received any in -services training regarding maternal and or child health services delivery in CDRO | 1. Yes 2. No |

**Part two: Items to assess community pharmacy professionals’ practice in responding to minor symptoms during pregnancy**

| 1. Considering your role in management of minor symptoms during pregnancy: How often do you gather the following symptom related information before providing a treatment? | | | | | |
| --- | --- | --- | --- | --- | --- |
| Items | | Response  Always=4, Often=3, Sometimes=2 and never=1 | | | |
| 1 | Duration of symptoms | Always | Often | Sometimes | Never |
| 2 | Frequency of symptoms | Always | Often | Sometimes | Never |
| 3 | Comorbidity | Always | Often | Sometimes | Never |
| 4 | Age of the woman | Always | Often | Sometimes | Never |
| 5 | Gestational age/Trimester/ | Always | Often | Sometimes | Never |
| 6 | Weight of the woman | Always | Often | Sometimes | Never |
| 7 | Previous medical conditions | Always | Often | Sometimes | Never |
| 8 | Previous medication history and current medication, allergy history | Always | Often | Sometimes | Never |
| 1. Considering your role in responding to symptoms for pregnant women: How often do you inform the patients about the following medication related information when dispensing a medication? | | | | | |
| 1 | Name of the medication | Always | Often | Sometimes | Never |
| 2 | Purpose/use of medication | Always | Often | Sometimes | Never |
| 3 | Dosage form | Always | Often | Sometimes | Never |
| 4 | Dose | Always | Often | Sometimes | Never |
| 5 | Information on how to use the medication and its application | Always | Often | Sometimes | Never |
| 6 | Duration of use | Always | Often | Sometimes | Never |
| 7 | Side effect | Always | Often | Sometimes | Never |
| 8 | Drug interaction | Always | Often | Sometimes | Never |
| 9 | Importance of compliance/adherence | Always | Often | Sometimes | Never |
| 10 | Storage conditions | Always | Often | Sometimes | Never |

**Part three: three: Items to assess the most common types of minor symptoms**

**and recommendations during pregnancy based on case study**

| Items | Response |
| --- | --- |
| 1. For which minor symptoms do customers who are pregnant visit your CDRO commonly?   **More than one answer is possible* | 1. Nausea and vomiting 2. Indigestion 3. Headache 4. Back pain 5. Cough 6. Constipation 7. Diarrhea 8. Vaginal itching and discharge 9. Common cold 10. Other (specify) |
| Case study: The responses to the following questions are based on specific case scenarios. The cases are designed to assess your practice in management of minor symptoms for pregnant women. | |
| Case 1: Back pain  A pregnant woman with 8^th^ month of pregnancy complaining of back pain comes to your pharmacy. She requests you to give her a medication for her back pain. Assume you are the one who is responsible to manage her case at the pharmacy. | |
| Question | Response |
| 1. What will be your decision regarding her request? | 1. Dispensing medication 2. Refer to hospital for further evaluation 3. Dispensing medication and refer to hospital for further evaluation 4. Don’t dispense any mediation and don’t advise to refer to hospital |
| 1. From the following, which information/s are you going to gather before providing the medication? (Assume that your decision is to give her a medication)   **More than one is possible* | 1. Duration of symptoms 2. Frequency of symptoms 3. Comorbidity 4. Age of the woman 5. Gestational age/Trimester/ 6. Weight of the woman 7. Previous medical conditions 8. Previous medication history and current medication, allergy Hx |
| 1. From the following medication related information, which information/s are you going to provide to her?   *More than one is possible* | 1. Name of the medication 2. Purpose of medication 3. Dosage form 4. Dose 5. Information on how to use the medication and its application 6. Duration of use 7. Side effect 8. Drug interaction 9. Importance of compliance/adherence 10. Storage conditions |
| Case 2: nausea and vomiting  A pregnant woman in the 9^th^ weeks of pregnancy complains of moderate nausea and vomiting comes to your pharmacy and requests you to provide medication to alleviate her symptoms. | |
| Question | Response |
| 1. What is your decision on her request? | 1. Dispensing medication 2. Refer to hospital for further evaluation 3. Dispensing medication and refer to hospital for further evaluation 4. Don’t dispense any mediation and don’t advise to refer to hospital |
| 1. From the following, which information/s are you going to gather before providing the medication? (Assume that your decision is to give her a medication)   *More than one is possible* | 1. Duration of symptoms 2. Frequency of symptoms 3. Comorbidity 4. Age of the woman 5. Gestational age/Trimester/ 6. Weight of the woman 7. Previous medical conditions 8. Previous medication history and current medication, allergy Hx |
| 1. From the following medication related information, which information/s are you going to provide to her?   *More than one is possible* | 1. Name of the medication 2. Purpose of medication 3. Dosage form 4. Dose 5. Information on how to use the medication and its application 6. Duration of use 7. Side effect 8. Drug interaction 9. Importance of compliance/adherence 10. Storage conditions |
