# Supplementary material for: Community pharmacy professionals’ practice in responding to minor symptoms experienced by pregnant women in Ethiopia: results from sequential mixed methods
Source: J Pharm Policy Pract. 2022 Apr 6;15:29. doi: 10.1186/s40545-022-00427-x (PMC8988357; doi:10.1186/s40545-022-00427-x)
Supplement: Supplementary file 2 — Additional file 2. Simulated client visit outcome recording forms. [file 40545_2022_427_MOESM2_ESM.docx]

**Additional file 2: Simulated client visit outcome recording forms**

**Simulated case scenario 1: Back pain**

1. Location of the Community Drug Retail Outlet (CDRO) …………………………………………………………….
2. Code of the CDRO ………………………………………………………………….
3. What was the decision of the community pharmacy professional?
4. Dispensing medication
5. Refer to hospital for further evaluation
6. Dispensing medication and refer to hospital for further evaluation
7. Didn’t dispense any mediation and didn’t advise to refer to hospital
8. Evaluate the points below regarding information asked about the case by the community pharmacy professional.

| Types of information gathered | Yes: Information is gathered  No: Information is not gathered | |
| --- | --- | --- |
|  | Yes | No |
| 1. Asked about duration of symptoms |  |  |
| 1. Asked about duration frequency of symptoms |  |  |
| 1. Comorbidity |  |  |
| 1. Age of the woman |  |  |
| 1. Asked about gestational age/Trimester/ |  |  |
| 1. Weight of the woman |  |  |
| 1. Previous medical conditions |  |  |
| 1. Previous medication history and current medication, allergy Hx |  |  |

1. Evaluate the points below regarding information provided by the community pharmacy professional

| Type of information provided | Yes: Provided No: Not provided | |
| --- | --- | --- |
|  | Yes | No |
| 1. Name of the medication |  |  |
| 1. Purpose of medication |  |  |
| 1. Dosage form |  |  |
| 1. Dose |  |  |
| 1. Information on how to use the medication and its application |  |  |
| 1. Duration of use |  |  |
| 1. Side effect |  |  |
| 1. Drug interaction |  |  |
| 1. Importance of compliance/adherence |  |  |
| 1. Storage conditions |  |  |

**Simulated case 2: Nausea and vomiting during**

1. What was the decision of the community pharmacy professional?
2. Dispensing medication
3. Refer to hospital for further evaluation
4. Dispensing medication and refer to hospital for further evaluation
5. Didn’t dispense any mediation and didn’t advise to refer to hospital
6. Evaluate the points below regarding information asked about the case by the community pharmacy professional.

| Types of information gathered | Yes: Information is gathered  No: Information is not gathered | |
| --- | --- | --- |
|  | Yes | No |
| 1. Asked about duration of symptoms |  |  |
| 1. Asked about duration frequency of symptoms |  |  |
| 1. Comorbidity |  |  |
| 1. Age of the woman |  |  |
| 1. Asked about gestational age/Trimester/ |  |  |
| 1. Weight of the woman |  |  |
| 1. Previous medical conditions |  |  |
| 1. Previous medication history and current medication, allergy Hx |  |  |

1. Evaluate the points below regarding information provided by the community pharmacy professional.

| Type of information provided | Yes: Provided No: Not provided | |
| --- | --- | --- |
|  | Yes | No |
| 1. Name of the medication |  |  |
| 1. Purpose of medication |  |  |
| 1. Dosage form |  |  |
| 1. Dose |  |  |
| 1. Information on how to use the medication and its application |  |  |
| 1. Duration of use |  |  |
| 1. Side effect |  |  |
| 1. Drug interaction |  |  |
| 1. Importance of compliance/adherence |  |  |
| 1. Storage conditions |  |  |
